# Clinical prediction models for the early diagnosis of obstructive sleep apnea in stroke patients: a systematic review

**DOI:** 10.1186/s13643-024-02449-9

**Published:** 2024-01-24

**Authors:** Hualu Yang, Shuya Lu, Lin Yang

**Affiliations:** 1 Department of Rehabilitation, Huazhong University of Science and Technology Union Shenzhen Hospital, Shenzhen, 581052 China; 2 School of Nursing, The Hong Kong Polytechnic University, Hong Kong SAR, 999077 China

**Keywords:** Obstructive sleep apnea, Stroke, Prediction models, Systematic review

## Abstract

**Background:**

Obstructive sleep apnea (OSA) is a common sleep disorder characterized by repetitive cessation or reduction in airflow during sleep. Stroke patients have a higher risk of OSA, which can worsen their cognitive and functional disabilities, prolong their hospitalization, and increase their mortality rates.

**Methods:**

We conducted a comprehensive literature search in the databases of PubMed, CINAHL, Embase, PsycINFO, Cochrane Library, and CNKI, using a combination of keywords and MeSH words in both English and Chinese. Studies published up to March 1, 2022, which reported the development and/or validation of clinical prediction models for OSA diagnosis in stroke patients.

**Results:**

We identified 11 studies that met our inclusion criteria. Most of the studies used logistic regression models and machine learning approaches to predict the incidence of OSA in stroke patients. The most frequently selected predictors included body mass index, sex, neck circumference, snoring, and blood pressure. However, the predictive performance of these models ranged from poor to moderate, with the area under the receiver operating characteristic curve varying from 0.55 to 0.82. All the studies have a high overall risk of bias, mainly due to the small sample size and lack of external validation.

**Conclusion:**

Although clinical prediction models have shown the potential for diagnosing OSA in stroke patients, their limited accuracy and high risk of bias restrict their implications. Future studies should focus on developing advanced algorithms that incorporate more predictors from larger and representative samples and externally validating their performance to enhance their clinical applicability and accuracy.

**Supplementary Information:**

The online version contains supplementary material available at 10.1186/s13643-024-02449-9.

## Background

Obstructive sleep apnea (OSA) is the most common sleep disorder, characterized by recurrent interruptions in breathing during sleep. Individuals with OSA often present clinical symptoms such as sleepiness, fatigue, and headache [1]. The incidence of OSA in stroke patients increased from 61% in 2011 to 75% in 2019 [2–4], a rate significantly higher than the 35% found in the general population [5]. Previous studies found that OSA was associated with prolonged hospital stay, increased recurrence of stroke, and elevated mortality rates among stroke patients [6–9]. Both the American Heart Association and the American Stroke Association recommend that the diagnosis and treatment of OSA should be part of secondary prevention programs for stroke [10]. Therefore, it is important to ensure that patients with OSA receive timely and effective diagnosis and treatment.

The polysomnography (PSG), conducted in a sleep laboratory by a trained physician, is widely recognized as the gold standard for OSA diagnosis [11]. Patients experiencing an average of at least 15 apnea events per hour are typically diagnosed with OSA [12]. However, due to high costs and significant manpower required for routine PSG screenings in clinical settings, the actual incidence of OSA is seriously underreported [13, 14]. A cross-sectional survey in the USA showed that only 5% of stroke patients took PSG examinations for OSA diagnosis [13]. Home sleep apnea testing (HSAT) is also recommended as an alternative diagnostic method, although it has slightly lower sensitivity than PSG [12]. Therefore, PSG is necessary for OSA diagnosis, particularly in patients who have negative HSAT results but present clinical symptoms of OSA [15]. Hence, studies have been conducted to develop convenient and accurate prediction models based on demographic and clinical characteristics for early identification of high-risk OSA [16].

Numerous screening tools for identifying the risk of OSA in stroke patients have been developed and validated, including the Berlin Questionnaire (BQ), Epworth Sleepiness Scale, four‐variable screening tool, and Sleep Apnea Clinical Score [17]. In this study, we conducted a systematic review of the performance of these prediction models and evaluated the feasibility of adopting these models for predicting OSA risk in stroke patients.

## Methods

This review was conducted following the guidelines of the Preferred Reporting Items for Systematic Reviews and Meta-Analyses (PRISMA) [18].

The inclusion criteria were as follows: (1) studies involving adults aged 18 years or above who were admitted for stroke, (2) studies focusing on prediction models for the early diagnosis of OSA, (3) studies on the development of a new prediction model for incident OSA with internal and/or external validations, and (4) studies that adopted the PSG or HSAT as the gold standard for OSA diagnosis for model internal and/or external validations. The studies were limited to those published in English and Chinese. There was no time restriction for the literature search. Secondary sources such as reviews or meta-analyses were excluded. No other exclusion criteria were applied in this review.

### Search strategy

We conducted the literature search on March 1, 2022, in the English database CINAHL, Embase, PsycINFO, and PubMed, as well as in the Chinese literature database CNKI.

Only articles published in English and Chinese were included. In addition, PhD dissertations and related articles were searched by using the Google Scholar. The reference lists of all selected studies were manually searched for additional literature. The MeSH terms and keywords used in the electronic search were {“obstructive sleep apnea” OR “obstructive sleep apnea syndrome” OR “sleep apnea hypopnea syndrome” OR "sleep apnea, obstructive" OR "sleep disordered breathing"} AND}“stroke” OR “cerebrovascular accident” OR “brain vascular accident” OR “acute stroke”} AND {“prediction” OR “predictor” OR "screening” OR “assess” OR “identify” OR “predictive value of test” OR “risk assessment” OR “risk factors” OR “questionnaire”}. Detailed English keywords and corresponding Chinese keywords are shown in Appendix 1.

### Study selection and screening

Two reviewers (H. Y. and S. L.) screened the title and abstracts of searched articles for relevancy. The methodological quality and risk of bias of each selected article were independently evaluated by the two reviewers using the Prediction model Risk-of-Bias Assessment Tool (PROBAST) [19]. This tool was used to identify the potential risk of the model on the basis of four domains: participant, predictor, outcome, and analysis. Each domain had two or more signal questions. If the response to one or more signal question was “no,” this domain was considered as high risk. If no information to answer to the question was available, this domain was considered as “unclear.” The third reviewer (L. Y.) participated in the discussion in the case of discrepancies to reach a consensus.

### Data extraction

A standardized form was used to tabulate the included articles and retrieved related information in accordance with the CHARMS checklist [20]. Two reviewers extracted relevant information from the selected literature independently by using the standardized data extraction form. A third reviewer was involved in discussions in case of discrepancies in extraction. The extracted study information included authors, years of publication, study design, participant characteristics (age, sample size, recruitment method, study period, settings, and stroke stage), outcome measured (method and time point of measurement), predictor (candidate predictor and final model predictor), method for handling missing data, model development (type of model, methods for selecting predictors, and model format), and model performance (calibration and discrimination).

## Results

### Characteristics of the included studies

**Fig. 1:**
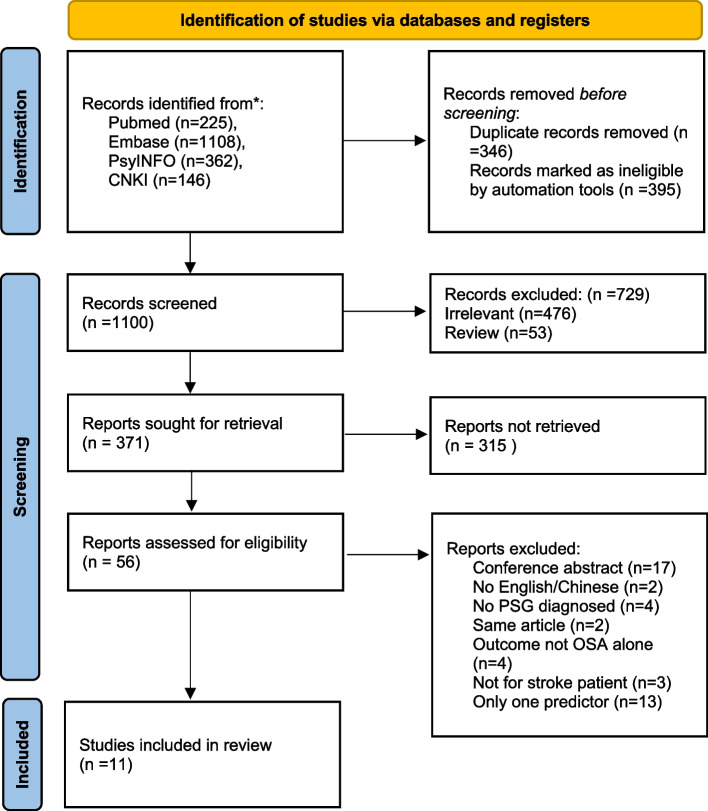
PRISMA flow chart of study selection

A total of 2874 records were identified through electronic databases and keyword searches. A total of 1931 articles remained after removing duplicates and screening titles and abstracts for eligibility. The guidelines of the critical appraisal and data extraction for systematic reviews of prediction modeling studies (CHARMS) checklist were used for assessment of the abstracts of identified articles [20]. Two reviewers independently screened the full texts of the remaining 101 articles for eligibility. Eleven studies were selected for this review (Fig. 1).

The characteristics of the 2837participants in these 11 studies are summarized in Table 1. The average age of the participants was 60.7 years [21–31]. The majority of participants (71.3%) were in the acute stage of stroke (less than 7 days), while 22.8% were in both the acute and subacute stages (less than 6 months). Almost 85% participants were from hospitals [21–23, 25–28, 30, 31], including neurology, stroke unit, or emergency units, while 15% were from stroke clinics [24, 29]. The studies were conducted in seven different countries: the USA, Canada, China, Brazil, Slovakia, India, and Italy. Most studies (72.7%) adopted a cross-sectional study design [21, 22, 24, 25, 28–31], while two studies were retrospective cohort studies [26, 27].

**Table 1:** Participant characteristics in the selected studies

**Author/year**	**Country**	**Data sources**	**Sample size**	**Age (years)**	**Eligibility criteria**	**Study period**	**Settings**	**Stroke stage**
Sico/2017 [23]	America	Randomized trials	303	Development: 70.8 ± 9.9 Validation: 60.7 ± 9.9	Patient with ischemic stroke; history with hypertension	2004–2008	Hospital	Acute or subacute
Brown/2020 [25]	America	Cross-sectional study	1330	65.0 ±12.6	Patients with stroke; ≥ 45 years old	2010–2018	Acute care hospital	Acute
Boulos/2019 [29]	Canada	Cross-sectional study	231	64.4 ± 15.3	Outpatients with ischemic Stroke; English speaking;	4/2011–7/2017	Stroke prevention clinic	No restriction
Katzan/2016 [27]	America	Retrospective cohort	208	55.4 ± 14.1	Patients with stroke	1/2011–12/2012	Cerebrovascular clinic	No restriction
Bernardini/2021 [21]	Italy	Cross-sectional study	30	Not mentioned	Patient with cerebrovascular event	8/2019–7/2020	Stroke unit	Acute
Zhang/2019 [22]	China	Cross-sectional study	124	62.6 ± 12.6	Patients with stroke	6/2016–5/2017	Neurology unit	Acute
Boulos/2016 [24]	Canada	Cross-sectional study	69	68.3 ± 14.2	Patients with stroke (ischemic or hemorrhagic)	7/2014–6/2015	Stroke unit or stroke prevention clinic	Acute and subacute
Petrie/2021 [26]	America	Retrospective cohort	344	59.0 ± 11.8	Patients with acute stroke, subarachnoid hemorrhage	10/2014–10/2015	Stroke unit	Acute
Šiarnik/2020 [31]	Slovakia	Cross-sectional study	120	Development: 67.2 ± 9.1 Validation: 62.4 ± 13.3	Patients with acute ischemic stroke	Not mentioned	Stroke unit	Acute
Camilo/2014 [28]	Brazil	Cross-sectional study	39	63.2 ± 12.2	Patients with first ischemic stroke > 18 years old	Not mentioned	Emergency unit	Acute
Srijithesh/2011 [30]	India	Cross-sectional study	39	56.5	Patients with hemorrhagic or ischemic stroke	Not mentioned	Neurology unit	Subacute

### Outcome variables and prediction factors

All the studies adopted diagnosed OSA as the outcome for prediction models. The diagnosis was based on either PSG or HSAT tests in sleep laboratories or at home, but the criteria varied among studies. Six studies defined the OSA as apnea hypopnea index (AHI) ≥ 10 times per hour [22, 24, 25, 27–29], four used the definition of *AHI* ≥ 5 times per hour [23, 26, 30], and one study adopted an AHI of ≥ 15 times per hour [29]. The time between the PSG test and stroke onset ranged from 1 day to 1 year. Most studies used PSG to test for OSA [21–23, 26–31], while others used HSAT [24, 25], and one study used both [29]. Five studies developed or updated a new model to predict the risk of OSA in stroke patients [21, 23, 25, 28, 31], three validated exiting models [22, 27, 29], and three developed and validated the same model [24, 26, 30].

The candidate predictors considered in these studies included demographics (age, gender, and race), clinical data (medical history, body mass index (BMI), blood pressure, waist circumference, and neck circumference, and disease severity measured by the National Institutes of Health Stroke Scale (NIHSS)), laboratory data (C-reactive protein, hemoglobin HbAlc, homocysteine, echocardiography, and oximetry), lifestyle factors (smoking, cocaine used, and alcohol consumption), sleep-related data (snoring, tiredness observed, Berlin Questionnaire (BQ), and Epworth Sleepiness Scale (ESS)), and wake-up stroke. The most commonly selected predictors in the models were BMI (*n* = 6), followed by sex (*n* = 5), neck circumference (*n* = 4), and snoring (*n* = 4). The other significant predictors included blood pressure, age, ESS, NIHSS, BQ, heart failure, and oximetry. Most predictors were obtained from medical records, such as demographic data, laboratory data, and anthropometric data, upon hospital admission. The interview time for sleep screening in four studies ranged from 1 night to 7 days after stroke onset [22, 24, 25, 29, 31], while the others did not specify a time point (Table 2).

**Table 2:** Outcome variables and predictors in the selected studies

Author/year	Outcome variables		Candidate predictors			Predictors in the final model	
	Time of assessment	Measurement method/definition	*N*	Variables	Time of assessment	*N*	Variables
Sico/2017 [23]	Within 30 days	PSG, AHI ≥ 5/h	19	Age, race, gender, height, weight, BMI, large neck circumference, waist circumference, medical history, smoking, cocaine use or used, Charlson comorbidity score, modified Rankin, PHQ-8, NIHSS score, ESS, BQ, SACS, STOP-BANG	No stated	7	Female, weight, large neck, congestive heart failure, diabetes, ESS, NIHSS
Brown/2020 [25]	Within 14 days from stroke	HSAT, REI ≥ 10/h	19	Age, race, hypertension, sex, diabetes, atrial fibrillation, smoking, history of TIA/stroke, excessive alcohol consumption, congestive heart failure, coronary artery disease, hyper cholesterol, BMI, NIHSS, neck circumference, waist circumference, BQ, Friedman palate position	After stroke onset	6	Neck circumference, BMI, waist circumference, age, NIHSS, daytime sleepiness
Boulos/2019 [29]				STOP-BAG (snoring, tired, observed, high blood pressure, BMI, age, neck circumference, gender)	After stroke diagnosis	8	STOP-BAG-O (snoring, tired observed, high blood pressure, BMI, age, neck circumference, gender, oximetry)
Katzan/2016 [27]				STOP (snoring, tired, observed, high blood pressure), BMI, age, race, neck circumference, married status, sex, history of coronary artery disease, sleep time, smoking	No stated	7	STOP-BAG2- (STOP, sex, BMI, age)
Bernardini/2021 [21]	No stated	PSG, AHI ≥ 5/h	No stated	No stated	No stated		ECG, peripheral oxygen saturation (SpO_2_)
Zhang/2019 [22]	No mentioned	PSG, AHI ≥ 10/h	11	Age, gender, BMI, neck circumference, smoking, alcohol consumption, medical history (hypertension, diabetes, AF, CHD, stroke, wake up stroke, progressive stroke, TIA, Ischemic stroke), NIHSS, homocysteine, CRP, hemoglobin A1c	With 1 week from stroke	4	Modified 4 V (sex, neck, blood pressure, snoring)
Boulos/2016 [24]	180 days after stroke	HSAT, AHI ≥ 10/h	10	4 V (sex, BMI, blood pressure, and snoring), STOP-BAG (snoring, tired, observed, high blood pressure, BMI, age, gender), Berlin Questionnaire, and ESS, stroke location, atrial fibrillation, diabetes, smoking, hyperlipidemia, NIHSS	Within 72 h of sleep testing	4	4 V (sex, BMI, blood pressure, snoring)
Petrie/2021 [26]	64 days after stroke	PSG, AHI ≥ 5/h	No fit	Not fit	After admission	/	/
Šiarnik/2020 [31]	Within 7 days after the stroke onset	PSG, AHI ≥ 15/h	12	Age, gender, BMI, neck, past medical history (arterial hypertension, diabetes mellitus, ischemic heart disease, atrial fibrillation, heart failure, chronic kidney disease), NIHSS, ESS	Within 7 days from stroke onset	3	SLAPS (BMI, wake-up stroke onset, diastolic dysfunction in echocardiography)
Camilo/2014 [28]	Within 24 h after stroke	PSG, AHI ≥ 10/h	13	Age, male, hypertension, diabetes, dyslipidemia, smoking, alcohol consumption, BMI, neck, BQ, CT, ESS, NIHSS	No stated	2	SOS score (BQ and ESS)
Srijithesh/2011 [30]	28 days after stroke onset	PSG, AHI ≥ 5/h	No stated	BQ	No stated	/	BQ and combination of ESS and BQ

*PSG* polysomnography, *HSAT* home sleep apnea test, *BMI* body mass index, *ESS* Epworth Sleepiness Scale, *NIHSS* National Institute of Health stroke scale, *PHQ-8* Patient Health Questionnaire, *CHD* coronary heart disease, *TIA* transient ischemic attack, *BQ* Berlin Questionnaire, *SACS* sleep apnea clinical score, *CT* brain computed tomography, *SOS* sleep obstructive apnea score, *AF* atrial fibrillation, *CRP* C-reactive protein, *4 V* the four-variable screening tool

### Model development and performance

Five studies developed or updated a new model to predict the risk of OSA in stroke patients, while three studies only validated the exiting models in different settings. Five of the developed models were logistic regression models [23, 24, 27, 29, 31], two adopted machine learning approaches such as random forest and convolutional neural network [21, 25], and one study simply combined variables from two existing instruments to validate its performance [30]. Four studies reported the predictor selection process, including backward selection [23], stepwise selection [25, 31], and bootstrapping [27], but none reported the model’s goodness of fit or calibration. The logistic regression models developed in these studies had low to moderate performance, with the area under the curve (AUC) ranging from 0.68 to 0.83 and specificity from 28 to 71.9% [22–24, 26, 27, 29–31] (Table 3). Four studies conducted internal validation [24, 27, 29, 31], but only one performed both internal and external validation [23].

**Table 3:** Modeling strategies adopted in the selected studies

**Author/year**	**Missing data**	**Model development**						**Model performance**
		Model type	Predictor selection method	Model format	Validation methods	Calibration	Discrimination	Classification
Sico/2017 [23]	No imputation	Logistic regression	Backward with uniform *P*-value	/	External validation	/	D-C: 0.732 V-C: 0.731	**D-**SN: 91.4%; SP: 43.8%; NPV: 76.2%, PPV: 72.1%; **V-**SN: 100%; SP: 12.5%; NPV: 100%; PPV: 79.6%
Brown/2020 [25]	Separate category by default	Machine learning	Stepwise selection	/	/	/	C: 0.75	/
Boulos/2019 [29]	/	Logistic regression	/	/	Bootstrapping	/	C: 0.751	SN: 95.9%; SP: 26.1%; PPV: 48.4%; NPV: 89.7%
Katzan/2016 [27]	Multiple imputation	Logistic regression	Bootstrapping	Formula	Bootstrapping	/	STOP-BAG2 + C: 0.84	SN: 94%; SP: 60%
Bernardini/2021 [21]	/	Convolutional deep learning	/	/	/	/	/	/
Zhang/2019 [22]	/	/	/	/	/	/	AUC: 0.835	SN: 74.1%; SP: 76.9%; PPV: 87.5%; NPV: 57.7%
Boulos/2016 [24]	/	Logistic regression	/	/	/	/	AUC: STOP-BAG: 0.677; 4 V: 0.688; BQ: 0.563; SOS: 0.506	4 V: SN: 59.4%; SP: 59.5%: PPV: 55.9%; NPV: 62.9%
Petrie/2021 [26]	/	/	/	/	/	/	C-statistic SB, 0.572; ESS, 0.502; BQ, 0.640	BQ: SN: 36%; SP: 62% ESS: SN: 68%; SP: 62% SB: SN: 81%; SP: **33**%
Šiarnik/2020 [31]	/	Logistic regression	Stepwise selection	/	/	/	AUC: 0.81	SN: 82.9%; SP: 71.9%,
Camilo/2014 [28]	/	Logistic regression	/	/	/	/	AUC: 0.813	SN: 90%; NPV: 94.5%; SP: 55.6%, PPV: 27.1%
Srijithesh/2011 [30]	/		/	/	/	/		BQ: SN: 68.2%, SP: 58.8%, PPV: 68.2%, NPV: 58.8% Combined BQ & ESS: SN: 50%, SP: 88.2%, PPV: 84.6%, NPV: 57.7%

*D* developed, *V* validated, *C* c-index, *AUC* area under the curve, *SN* sensitivity, *SP* specificity, *NPV* negative predictive value, *PPV* positive predictive value,

*SB* STOP-BANG (snoring, tired, observed, high blood pressure, BMI, age, neck circumference, gender), *4 V* the four-variable screening tool, *STOP-BAG* snoring, tired, observed, high blood pressure, BMI, age, gender, *BQ* Berlin Questionnaire, *SOS* sleep obstructive apnea score, *ESS* Epworth Sleepiness Scale

### Quality assessment

Based on the PROBAST criteria, the participant selection domain was rated as low risk of bias, as all studies adopted appropriate study design and inclusion/exclusion criteria (Table 4). However, the application of this domain was judged as high concern, as four studies also included patients diagnosed with transient ischemic attack [23, 24, 26, 29]. The predictor domain was assessed as high risk of bias, because three studies did not clearly state whether predictors were measured in the same way [22, 23, 29]. The application of this domain was judged as high concern due to inconsistent predictor assessment times and unclear predictor measurement methods. All studies defined OSA diagnosis based on the PSG test; hence, the risk of bias and application concern in the outcome domain were low. The analysis domain was rated as high risk of bias, as all the included studies assessed the models’ discrimination or classification performance, but none described model calibration. Only three studies used appropriate methods to handle missing data [23, 27, 29]. The methods used to handle of missing data by other models were unclear. The overall risk of bias and application concern were high in these studies (Table 5).

**Table 4:** Quality assessment of the selected studies by Prediction model Risk-of-Bias Assessment Tool (PROBAST)

Author/year	Risk of bias				Application			Overall assessment	
	**Participants**	**Predictors**	**Outcomes**	**Analysis**	**Participants**	**Predictors**	**Outcomes**	**ROB**	**Applicability**
Sico/2017 [23]	+	-	+	−	−	−	+	−	−
Brown/2020 [25]	+	+	+	−	+	+	+	−	−
Boulos/2019 [29]	+	-	+	−	−	+	+	−	−
Katzan/2016 [27]	+	+	+	−	+	−	+	−	−
Bernardini/2021 [21]	+	+	+	−	+	−	+	−	−
Zhang/2019 [22]	+	-	+	−	+	+	+	−	+
Boulos/2016 [24]	+	+	+	−	−	+	+	−	−
Petrie/2021 [26]	+	+	+	−	−	+	+	−	−
Šiarnik/2020 [31]	+	+	+	−	+	+	+	−	+
Camilo/2014 [28]	+	+	+	−	+	−	+	−	−
Srijithesh/2011 [30]	+	+	+	−	+	+	+	−	+

+ Low risk of bias

− High risk of bias

?Unclear

**Table 5:** Quality assessment of the selected studies by Prediction model Risk-of-Bias Assessment Tool (PROBAST) (detailed)

Items		Author/year										
		Sico/2017	Brown/2020	Boulos/2019	Katzan/2016	Bernardini/2021	Zhang/2019	Boulos/2016	Petrie/2021	Šiarnik/2020	Camilo/2014	Srijithesh/2011
Participants	Were appropriate data sources used, e.g., cohort, randomized controlled trial, or nested case–control study data?	+	+	+	+	+	+	+	+	+	+	+
	Were all inclusions and exclusions of participants appropriate?	+	+	+	+	+	+	+	+	+	+	+
Predictors	Were predictors defined and assessed in a similar way for all participants?	+	+	?	+	+	?	+	+	+	+	+
	Were predictor assessments made without knowledge of outcome data?	?	+	?	+	+	?	+	+	+	+	+
	Are all predictors available at the time the model is intended to be used?	+	+	+	+	+	+	+	+	+	+	+
Outcome	Was the outcome determined appropriately?	+	+	+	+	+	+	+	+	+	+	+
	Was a prespecified or standard outcome definition used?	+	+	+	+	+	+	+	+	+	+	+
	Were predictors excluded from the outcome definition?	+	+	+	+	+	+	+	+	+	+	+
	Was the outcome defined and determined in a similar way for all participants?	+	+	+	+	+	+	+	+	+	+	+
	Was the outcome determined without knowledge of predictor information?	+	+	+	+	+	+	+	+	+	+	+
	Was the time interval between predictor assessment and outcome determination appropriate?	+	+	+	+	+	+	+	+	+	+	+
Analysis	Were there a reasonable number of participants with the outcome?	-	-	+	+	-	-	-	+	+	-	-
	Were continuous and categorical predictors handled appropriately?	+	+	+	+	+	+	+	+	+	+	+
	Were all enrolled participants included in the analysis?	+	+	+	+	+	+	+	+	+	+	+
	Were participants with missing data handled appropriately?	+	?	?	+	?	+	?	?	?	?	?
	Was selection of predictors based on univariable analysis avoided? (Model development studies only)	+	+	+	+	+	+	+	+	+	+	+
	Were complexities in the data (e.g., censoring, competing risks, sampling of control participants) accounted for appropriately?	+	+	+	+	+	+	+	+	+	+	+
	Were relevant model performance measures evaluated appropriately?	-	-	-	-	-	-	-	-	-	-	-
	Were model overfitting and optimism in model performance accounted for? (Model development studies only)	-	-	-	?	-	-	-	-	-	-	-
	Do predictors and their assigned weights in the final model correspond to the results from the reported multivariable analysis? (Model development studies only)?	-	-	-	+	-	-	-	-	-	-	-

+ Low risk of bias

− High risk of bias

?Unclear

## Discussion

Several models have been developed to assess the risk of OSA in the general population [32] or in patients with specific diseases such as spinal cord injury [33], pulmonary arterial hypertension [34], and diabetes [35]. However, these models may not be suitable for stroke patients. To identify stroke patients at high risk of OSA, 11 studies have been conducted. Only five of these studies proposed new models, while the rest either modified or validated existing models that were originally developed for the general population.

The models developed for predicting OSA in stroke patients exhibited low to moderate performance, with a high risk of bias observed during quality assessment. Developing an accurate prediction model for OSA in stroke patients is challenging. Common predictors like waist and neck circumference may be difficult to obtain in acute stroke patients, and some predictors adopted for the general population, such as observed tiredness, may not be applicable to stroke patients due to their similarity to stroke symptoms. Moreover, most studies had small sample sizes, particularly in acute patients who need emergency care, and the risk of OSA was often overlooked in this group. However, early diagnosis of OSA in acute stroke patients is crucial for their full recovery [36]. Therefore, there is an urgent need for modeling studies with larger sample sizes and routine collection of electronic medical datasets to develop valid and accurate prediction tools for identifying the risk of OSA among vulnerable stroke patients.

Similar to the models developed for the general population, OSA prediction models for stroke patients also selected predictors such as BMI, snoring, neck circumference, waist circumference, and hypertension. However, the data collection methods in these studies were not clearly specified, and predictors like neck circumference and waist circumference may not be easily available in acute stroke patients who are critically ill. In developing countries, a lack of assistive devices may further hinder the objective collection of data in stroke units, and staff often relies on patients or family members for such data. Therefore, it is crucial to include objective and readily available predictors for better predictive model performance, such as inflammatory biomarkers interleukin-6 (IL-6) or C-reactive protein (CRP), which have been shown to be related to an increase in OSA in stroke patients in previous studies [37, 38]. While oximetry and fatigue were utilized as predictors in some studies, these symptoms are similar to those of stroke disease, which could limit the model’s performance. Other valuable predictors such as infarct location [39], dysphagia [40], and nocturia [41], which have also been associated with OSA in stroke patients, require further exploration. Therefore, future research should incorporate the following predictors in the model: demographics such as age, gender, history of diabetes, smoking, and alcohol consumption; physical examination such as BMI, blood pressure, waist, and neck circumference; clinical data such as CRP, infarct location, and heart failure; sleep characteristic such as snoring, stop breathing, and ESS; and symptoms or severity associated with stroke such as dysphagia, nocturia, and NIHSS. Moreover, the objective and clinically assessable measurements of individual predictors are important. For example, dysphagia could be measured by various methods, such as Kubota water swallowing test (KWST), Gugging Swallowing Screen (GSS), fiber-optic endoscopic evaluation of swallowing (FEES), and ultrasound examination. KWST is commonly employed in clinical settings, but its specificity is suboptimal [42]. FEES is considered the preferred method for diagnosing swallowing disorders, but its application is restricted due to its invasive nature and associated high expenses [43]. Hence, ultrasound testing has become more commonly adopted for dysphagia diagnosis, owing to its lower cost and noninvasive nature [44].

Most of the studies included in this review were conducted in acute hospitals, with only a few conducted in primary care settings. It is worth noting that the prevalence of OSA was found to be higher in the acute phase of stroke (71.3%) compared to the chronic phase (60.6%) in a meta-analysis [45]. The differences in PSG test time across the included studies could have contributed to the poor performance of the prediction models. Furthermore, it was observed in clinical practice that individuals diagnosed with OSA as negative during the acute stage became positive during the chronic stage. In this review, the PSG test time varied from less than 24 h to 1 year after stroke onset, emphasizing the need for specific and standardized testing times. In future studies, separate prediction models for acute and chronic phases should be constructed to improve their clinical applicability.

In this systematic review, most studies used logistic regression for model construction, while a few also utilized deep learning or other machine learning algorithms. Due to the heterogeneity of included studies in the systematic reviews, there is no solid evidence to suggest differences between regression models and other machine learning models. However, one study of this review [25] showed that there was no significant difference in performance between machine learning and logistic regression models for stroke patients. Future studies should use various methods to develop models within the same populations and compare the effectiveness of these different approaches. This would provide more valuable guidance that could be beneficial to clinical practice.

In addition, the selection of a model should also take into account factors such as the sample size, the nature of data, and the purpose of model construction [46]. For instance, logistic regression is a common statistical method known for its simplicity and interpretability, frequently used in developing prediction models. However, it requires a clear structural relationship between outcome variables and predictors [25]. The decision tree algorithm has high computational efficiency, making it suitable for small datasets with diverse data types [47]. The random forest model, which predicts by aggregating the outcomes of numerous recursively partitioned tree models, is suitable for constructing supervised models with large sample sizes [48].

Although numerous studies have developed or validated prediction models for stroke patients, the generalization of these models was poor due to the lack of external validation. Of the 11 studies included in the review, only three reported the process of internal validation, and only one performed external validation [23]. Additionally, the absence of detailed algorithms hinders the external validation of these models. None of the studies included in the review reported applying their models to online accessible risk calculation tools, despite their potential benefit for stroke patients. It is of note that this review might have missed some studies published in languages other than English and Chinese due to language restrictions. Nevertheless, future research should focus on strengthening external validation tests and selecting appropriate methods to validate models, to improve the generalization of these models.

In this review, the quality of the included studies was found at high risk of bias in terms of study design, predictors, and the handling of missing data. The majority of studies adopted cross-sectional design, which may be suitable for diagnostic models but not for the early prediction of OSA incidence [49]. Furthermore, the included studies poorly reported the number and handling method of missing data. Only three studies reported the detailed process of predictor selection, and stepwise selection, a widely used traditional method, was employed. However, previous evidence has confirmed that stepwise selection could generate the risk of model overfitting [50]. Modern statistical methods, such as bootstrapping or the least absolute shrinkage and selection operator, are promising methods for identifying important variables to resolve the overfitting problem [51, 52]. Therefore, future studies should restrict the candidate list and adopt the shrinkage method to develop high-quality prediction models.

In this review, model discrimination performance, as indicated by AUC values, varied from 0.502 to 0.84, with newly developed models performing better than existing models for the general population. Future models should consider incorporating factors related to patients with stroke to enhance their quality. Calibration, defined as the agreement between observed outcome and prediction, is also important, but none of the included studies in this review described it using the calibration plot or Hosmer–Lemeshow test [27]. Therefore, future studies should include calibration and discrimination in assessing model clinical usefulness. Additionally, model classification largely depends on the predefined threshold and should be carefully considered based on clinical settings in future studies.

Given the high prevalence of undiagnosed OSA in the general population [53, 54], it is crucial to develop advanced tools that can effectively identify individuals at high risk. These tools should help healthcare professionals and patients make informed decisions, streamline the referral process for PSG testing, ensure accurate diagnoses, and promote prompt initiation of treatment. Various scales, such as the Berlin Questionnaire, STOP-BANG, and ESS, have been commonly used, along with regression models, to detect high-risk populations. For instance, Chang et al. utilized snoring in sitting as predictors, while the OSA50 scale incorporated age 50 or older, snoring, observed apnea, and waist circumference for predictions [55]. Other studies have included tongue position, BMI, and tonsil size as predictors [56]. However, these models have shown low to moderate performance. Dysphagia, a symptom frequently observed in stroke patients, impacts an estimated 38.5 to 50% of individuals who have experienced a stroke [57, 58]. Previous research has indicated that dysphagia serves as an independent risk factor for stroke patients with OSA [59]. Additionally, the location of the infarction within the brain stem has been associated with the severity of OSA in stroke patients [60]. These identified predictors could be integrated into forthcoming models as stroke-specific factors, thereby enhancing the efficacy and precision of these predictive models. Furthermore, in terms of model development methods, exploring artificial intelligence models like random forests and decision trees in the general population are necessary. Regarding the application of the model, utilizing web-based methods to present the developed model can enhance its applicability and assist clinical medical personnel, family caregivers, or individuals themselves in early screening.

## Conclusion

Various prediction models for OSA in stroke patients have been developed or validated, but their performance was found to be low, and the methodology had high-risk bias. To address these issues, future studies should focus on the following gaps: first, successful prediction models for stroke patients should incorporate accessible clinical predictors. Second, internal and external validation should be conducted using a sufficient sample size, and missing values should be appropriately handled to reduce bias. Providing an easily accessible final model for clinical work, such as through web-based calculators or apps, is valuable. Additionally, subgroup comparisons, such as patients with acute, subacute, or chronic stroke, should be taken into account. Finally, generalization can be increased by collecting samples from multiple centers and different environments.

### Supplementary Information

**Additional file 1:** **Appendix 1.** PubMed (-2022/03/01). EMBASE (-2022/03/01). PsycINFO(-2022/03/01).

## Data Availability

Not applicable.

## Abbreviations

OSA: Obstructive sleep apnea
PSG: Polysomnography
BQ: Berlin Questionnaire
PRISMA: Preferred Reporting Items for Systematic Reviews and Meta-Analyses
HSAT: Home sleep apnea test
CHARMS: Critical appraisal and data extraction for systematic reviews of prediction modeling studies
AHI: Apnea hypopnea index
BMI: Body mass index
NIHSS: National Institutes of Health Stroke Scale
ESS: Epworth Sleepiness Scale
AUC: Area under the curve

## References

[1] Jordan AS, Mcsharry DG, Malhotra A (2014). Adult obstructive sleep apnoea. Lancet.

[2] Hermann DM, Bassetti CL (2009). Sleep-related breathing and sleep-wake disturbances in ischemic stroke. Neurology.

[3] Johnson KG, Johnson DC (2010). Frequency of sleep apnea in stroke and TIA patients: a meta-analysis. J Clin Sleep Med.

[4] Brown DL, Gibbs R, Shi X, Case E, Chervin R, Lisabeth LD (2021). Growing prevalence of post-stroke sleep-disordered breathing. Stroke.

[5] Ghavami T, Kazeminia M, Ahmadi N, Rajati F (2023). Global prevalence of obstructive sleep apnea in the elderly and related factors: a systematic review and meta-analysis study. J Perianesth Nurs.

[6] King S, Cuellar N (2016). Obstructive sleep apnea as an independent stroke risk factor: a review of the evidence, stroke prevention guidelines, and implications for neuroscience nursing practice. J Neurosci Nurs..

[7] Mckee Z, Auckley DH (2019). A sleeping beast: obstructive sleep apnea and stroke. Cleve Clin J Med..

[8] Chen CY, Chen CL (2021). Recognizable clinical subtypes of obstructive sleep apnea after ischemic stroke: a cluster analysis. Nat Sci Sleep..

[9] Zhang Y, Wang W, Cai S, Sheng Q, Pan S, Shen F, Tang Q, Liu Y (2017). Obstructive sleep apnea exaggerates cognitive dysfunction in stroke patients. Sleep Med.

[10] Hemphill JC 3rd, Greenberg SM, Anderson CS, Becker K, Bendok BR, Cushman M, Fung G L, Goldstein JN, Macdonald RL, Mitchell PH, Scott PA, Selim MH, Woo D. American heart association stroke council, council on cardiovascular and stroke nursing, & council on clinical cardiology. Guidelines for the management of spontaneous intracerebral hemorrhage: A guideline for healthcare professionals from the American heart association/American stroke association. Stroke. 2015;46(7):2032–60. 10.1161/STR.0000000000000069. 10.1161/STR.0000000000000069 26022637

[11] Ichikawa M, Akiyama T, Tsujimoto Y, Anan K, Yamakawa T, Terauchi Y (2022). Diagnostic accuracy of home sleep apnea testing using peripheral arterial tonometry for sleep apnea: a systematic review and meta-analysis. J Sleep Res.

[12] Kapur VK, Auckley DH, Chowdhuri S, Kuhlmann DC, Mehra R, Ramar K, Harrod CG (2017). Clinical practice guideline for diagnostic testing for adult obstructive sleep apnea: an American Academy of Sleep Medicine clinical practice guideline. J Clin Sleep Med.

[13] Brown DL, Jiang X, Li C, Case E, Sozener CB, Chervin RD, Lisabeth LD (2019). Sleep apnea screening is uncommon after stroke. Sleep Med.

[14] Festic N, Alejos D, Bansal V, Mooney L, Fredrickson PA, Castillo PR, Festic E (2018). Sleep apnea in patients hospitalized with acute ischemic stroke: underrecognition and associated clinical outcomes. J Clin Sleep Med.

[15] Rosen CL, Auckley D, Benca R, Foldvary-Schaefer N, Iber C, Kapur V, Rueschman M, Zee P, Redline S (2012). A multisite randomized trial of portable sleep studies and positive airway pressure autotitration versus laboratory-based polysomnography for the diagnosis and treatment of obstructive sleep apnea: the HomePAP study. Sleep.

[16] Steyerberg EWJJotRSS. Clinical prediction models: a practical approach to development, validation, and updating by Ewout W. Steyerberg. 2010;66(2):661–2.

[17] Takala M, Puustinen J, Rauhala E, Holm A (2018). Pre-screening of sleep-disordered breathing after stroke: a systematic review. Brain Behav..

[18] Page MJ, McKenzie JE, Bossuyt PM, Boutron I, Hoffmann TC, Mulrow CD, Shamseer L, Tetzlaff JM, Moher D (2021). Updating guidance for reporting systematic reviews: development of the PRISMA 2020 statement. J Clin Epidemiol.

[19] Wolff RF, Moons KGM, Riley RD, Whiting PF, Westwood M, Collins GS, Reitsma JB, Kleijnen J, Mallett S (2019). PROBAST: a tool to assess the risk of bias and applicability of prediction model studies. Ann Intern Med.

[20] Moons KG, de Groot JA, Bouwmeester W, Vergouwe Y, Mallett S, Altman DG, Reitsma JB, Collins GS (2014). Critical appraisal and data extraction for systematic reviews of prediction modelling studies: the CHARMS checklist. PLoS Med.

[21] Bernardini A, Brunello A, Gigli GL, Montanari A, Saccomanno N (2021). AIOSA: an approach to the automatic identification of obstructive sleep apnea events based on deep learning. Artif Intell Med.

[22] Zhang L, Zeng T, Gui Y, Sun Y, Xie F, Zhang D, Hu X (2019). Application of neck circumference in four-variable screening tool for early prediction of obstructive sleep apnea in acute ischemic stroke patients. J Stroke Cerebrovasc Dis.

[23] Sico JJ, Yaggi HK, Ofner S, Concato J, Austin C, Ferguson J, Qin L, Tobias L, Taylor S, Vaz Fragoso CA (2017). Development, validation, and assessment of an ischemic stroke or transient ischemic attack-specific prediction tool for obstructive sleep apnea. J Stroke Cerebrovasc Dis.

[24] Boulos MI, Wan A, Im J, Elias S, Frankul F, Atalla M, Black SE, Basile VS, Sundaram A, Hopyan JJ (2016). Identifying obstructive sleep apnea after stroke/TIA: evaluating four simple screening tools. Sleep Med.

[25] Brown DL, He K, Kim S, Hsu CW, Case E, Chervin RD, Lisabeth LD (2020). Prediction of sleep-disordered breathing after stroke. Sleep Med.

[26] Petrie BK, Sturzoiu T, Shulman J, Abbas S, Masoud H, Romero JR, Filina T, Nguyen TN, Lau H, Clark J (2021). Questionnaire and portable sleep test screening of sleep disordered breathing in acute stroke and TIA. J Clin Med.

[27] Katzan IL, Thompson NR, Uchino K, Foldvary-Schaefer N (2016). A screening tool for obstructive sleep apnea in cerebrovascular patients. Sleep Med.

[28] Camilo MR, Sander HH, Eckeli AL, Fernandes RMF, dos Santos-Pontelli TEG, Leite JP, Pontes-Neto OM (2014). SOS score: an optimized score to screen acute stroke patients for obstructive sleep apnea. Sleep Med.

[29] Boulos MI, Colelli DR, Vaccarino SR, Kamra M, Murray BJ, Swartz RH (2019). Using a modified version of the “STOP-BANG” questionnaire and nocturnal oxygen desaturation to predict obstructive sleep apnea after stroke or TIA. Sleep Med.

[30] Srijithesh PR, Shukla G, Srivastav A, Goyal V, Singh S, Behari M (2011). Validity of the Berlin Questionnaire in identifying obstructive sleep apnea syndrome when administered to the informants of stroke patients. J Clin Neurosci.

[31] Šiarnik P, Jurík M, Klobučníková K, Kollár B, Pirošová M, Malík M, Turčáni P, Sýkora M (2021). Sleep apnea prediction in acute ischemic stroke (SLAPS score): a derivation study. Sleep Med.

[32] Park DY, Kim JS, Park B, Kim HJ (2021). Risk factors and clinical prediction formula for the evaluation of obstructive sleep apnea in Asian adults. PLoS ONE.

[33] Graco M, Schembri R, Cross S, Thiyagarajan C, Shafazand S, Ayas NT, Nash MS, Vu VH, Ruehland WR, Chai-Coetzer CL (2018). Diagnostic accuracy of a two-stage model for detecting obstructive sleep apnoea in chronic tetraplegia. Thorax.

[34] Hu M, Duan A, Huang Z, Zhao Z, Zhao Q, Yan L, Zhang Y, Li X, Jin Q, An C (2022). Development and validation of a nomogram for predicting obstructive sleep apnea in patients with pulmonary arterial hypertension. Nat Sci Sleep.

[35] Shi H, Xiang S, Huang X, Wang L, Hua F, Jiang X (2020). Development and validation of a nomogram for predicting the risk of obstructive sleep apnea in patients with type 2 diabetes. Ann Transl Med.

[36] Sanchez O, Adra N, Chuprevich S, Attarian H (2022). Screening for OSA in stroke patients: the role of a sleep educator. Sleep Med.

[37] Kunz AB, Kraus J, Young P, Reuss R, Wipfler P, Oschmann P, Blaes F, Dziewas R (2012). Biomarkers of inflammation and endothelial dysfunction in stroke with and without sleep apnea. Cerebrovasc Dis (Basel, Switzerland).

[38] Medeiros CA, de Bruin VM, Andrade GM, Coutinho WM, de Castro-Silva C, de Bruin PF (2012). Obstructive sleep apnea and biomarkers of inflammation in ischemic stroke. Acta Neurol Scand.

[39] Stahl SM, Yaggi HK, Taylor S, Qin L, Ivan CS, Austin C, Ferguson J, Radulescu R, Tobias L, Sico J (2015). Infarct location and sleep apnea: evaluating the potential association in acute ischemic stroke. Sleep Med.

[40] Shepherd K, Walsh J, Maddison K, Hillman D, McArdle N, Baker V, King S, Al-Obaidi Z, Bamagoos A, Parry R, et al. Dysphagia as a predictor of sleep-disordered breathing in acute stroke. J Sleep Res. 2018;27. 10.1111/jsr.13179 32856372

[41] Chen CY, Hsu CC, Pei YC, Yu CC, Chen YS, Chen CL (2011). Nocturia is an independent predictor of severe obstructive sleep apnea in patients with ischemic stroke. J Neurol.

[42] Tohara H, Saitoh E, Mays KA, Kuhlemeier K, Palmer JB (2003). Three tests for predicting aspiration without videofluorography. Dysphagia.

[43] Miller CK, Schroeder JW, Langmore S (2020). Fiberoptic endoscopic evaluation of swallowing across the age spectrum. Am J Speech Lang Pathol.

[44] Allen JE, Clunie GM, Winiker K (2021). Ultrasound: an emerging modality for the dysphagia assessment toolkit?. Curr Opin Otolaryngol Head Neck Surg.

[45] Liu X, Lam DC, Chan KPF, Chan HY, Ip MS, Lau KK (2021). Prevalence and determinants of sleep apnea in patients with stroke: a meta-analysis. J Stroke Cerebrovasc Dis.

[46] Sarker IH (2021). Machine learning: algorithms, real-world applications and research directions. SN Comp Sci.

[47] Song YY, Lu Y (2015). Decision tree methods: applications for classification and prediction. Shanghai Arch Psychiatry.

[48] Nguyen JM, Jézéquel P, Gillois P, Silva L, Ben Azzouz F, Lambert-Lacroix S, Juin P, Campone M, Gaultier A, Moreau-Gaudry A (2021). Random forest of perfect trees: concept, performance, applications and perspectives. Bioinformatics (Oxford, England).

[49] Steyerberg EW. Clinical prediction models: A practical approach to development, validation, and updating. AM J Epidemiol. 2009;170(4):528.

[50] Steyerberg EW, Uno H, Ioannidis JPA, van Calster B (2018). Poor performance of clinical prediction models: the harm of commonly applied methods. J Clin Epidemiol.

[51] Austin PC, Tu JV. Bootstrap methods for developing predictive models. Am Stat. 2004;58(2):131–7.

[52] Mallick H, Alhamzawi R, Paul E, Svetnik V (2021). The reciprocal Bayesian LASSO. Stat Med.

[53] Suen C, Wong J, Ryan CM, Goh S, Got T, Chaudhry R, Lee DS, Chung F (2020). Prevalence of undiagnosed obstructive sleep apnea among patients hospitalized for cardiovascular disease and associated in-hospital outcomes: a scoping review. J Clin Med.

[54] Del Campo F, Arroyo CA, Zamarrón C, Álvarez D (2022). Diagnosis of obstructive sleep apnea in patients with associated comorbidity. Adv Exp Med Biol.

[55] Chai-Coetzer CL, Antic NA, Rowland LS, Catcheside PG, Esterman A, Reed RL, Williams H, Dunn S, McEvoy RD (2011). A simplified model of screening questionnaire and home monitoring for obstructive sleep apnoea in primary care. Thorax.

[56] Friedman M, Wilson MN, Pulver T, Pandya H, Joseph NJ, Lin HC, Chang HW (2010). Screening for obstructive sleep apnea/hypopnea syndrome: subjective and objective factors. Otolaryngol Head Neck Surg.

[57] Carrión S, Cabré M, Monteis R, Roca M, Palomera E, Serra-Prat M, Rofes L, Clavé P (2015). Oropharyngeal dysphagia is a prevalent risk factor for malnutrition in a cohort of older patients admitted with an acute disease to a general hospital. Clin Nutr (Edinburgh, Scotland).

[58] Flamand-Roze C, Cauquil-Michon C, Denier C (2012). Tools and early management of language and swallowing disorders in acute stroke patients. Curr Neurol Neurosci Rep.

[59] Martínez-García MA, Galiano-Blancart R, Soler-Cataluña JJ, Cabero-Salt L, Román-Sánchez P (2006). Improvement in nocturnal disordered breathing after first-ever ischemic stroke: role of dysphagia. Chest.

[60] Brown DL, McDermott M, Mowla A, De Lott L, Morgenstern LB, Kerber KA, Hegeman G, Smith MA, Garcia NM, Chervin RD (2014). Brainstem infarction and sleep-disordered breathing in the BASIC sleep apnea study. Sleep Med.

