# Supplementary material for: Clinical prediction models for the early diagnosis of obstructive sleep apnea in stroke patients: a systematic review
Source: Syst Rev. 2024 Jan 24;13:38. doi: 10.1186/s13643-024-02449-9 (PMC10807185; doi:10.1186/s13643-024-02449-9)
Supplement: Supplementary file 1 — Additional file 1: Appendix 1. PubMed (-2022/03/01). EMBASE (-2022/03/01). PsycINFO(-2022/03/01). [file 13643_2024_2449_MOESM1_ESM.docx]

**PubMed (-2022/03/01)**

| **#** | **Search** | **Results** |
| --- | --- | --- |
| 1 | "Sleep Apnea, Obstructive"[Mesh] | 25051 |
| 2 | (obstructive sleep apnea[Title/Abstract]) OR (obstructive sleep apnea syndrome[Title/Abstract]) OR (sleep apnea hypopnea syndrome[Title/Abstract]) OR (sleep apnea, obstructive[Title/Abstract]) OR (sleep disordered breathing[Title/Abstract]) | 32806 |
| 3 | #1 OR #2 | 39656 |
| 4 | "Stroke"[Mesh] | 159540 |
| 5 | (stroke[Title/Abstract]) OR (cerebrovascular accident[Title/Abstract]) OR (brain vascular accident[Title/Abstract]) OR (acute stroke[Title/Abstract]) | 285576 |
| 6 | #4 OR #5 | 324788 |
| 7 | (Prediction[Title/Abstract]) OR (predictor[Title/Abstract]) OR (screening[Title/Abstract]) OR (assess[Title/Abstract]) OR (identify[Title/Abstract]) OR (predictive value of test[Title/Abstract]) OR (risk assessment[Title/Abstract]) OR (risk factors[Title/Abstract]) OR (questionnaire[Title/Abstract]) | 3862903 |
| 8 | #3 AND #6 AND #7 | 602 |

**E****MBASE (-2022/03/01)**

| **#** | **Search** | **Results** |
| --- | --- | --- |
| 1 | 'sleep disordered breathing'/exp | 89276 |
| 2 | 'obstructive sleep apnea':ab,ti OR 'obstructive sleep apnea syndrome':ab,ti OR 'sleep apnea hypopnea syndrome':ab,ti OR 'sleep apnea, obstructive':ab,ti OR 'sleep disordered breathing':ab,ti | 52116 |
| 3 | #1 OR #2 | 92374 |
| 4 | 'cerebrovascular accident'/exp | 366870 |
| 5 | stroke:ab,ti OR 'cerebrovascular accident':ab,ti OR 'brain vascular accident':ab,ti OR 'acute stroke':ab,ti | 433384 |
| 6 | #4 OR #5 | 537208 |
| 7 | prediction:ab,ti OR predictor:ab,ti OR screening:ab,ti OR assess:ab,ti OR identify:ab,ti OR 'predictive value of test':ab,ti OR 'risk assessment':ab,ti OR 'risk factors':ab,ti OR 'questionnaire':ab,ti | 5238594 |
| 8 | #3 AND #6 AND #7 | 2022 |

**PsycINFO(-2022/03/01)**

| **#** | **Search** | **Results** |
| --- | --- | --- |
| 1 | mainsubject.Exact("sleep apnea, obstructive") | ‎1675 |
| 2 | tiab(obstructive sleep apnea) OR tiab(obstructive sleep apnea syndrome) OR tiab(sleep apnea hypopnea syndrome) OR tiab(sleep apnea, obstructive) OR tiab(sleep disordered breathing) | 4133 |
| 3 | #1 OR #2 | 4289 |
| 4 | mainsubject.Exact("Stroke") | 12033 |
| 5 | tiab(stroke) OR tiab(cerebrovascular accident) OR tiab(brain vascular accident) OR tiab(acute stroke) | 34135 |
| 6 | #4 OR #5 | 35191 |
| 7 | tiab(prediction) OR tiab(predictor) OR tiab(screening) OR tiab(assess) OR tiab(identify) OR tiab(predictive value of test) OR tiab(risk assessment) OR tiab(risk factors) OR tiab(questionnaire) | 862917 |
| 8 | #3 AND #6 AND #7 | 104 |
